# Utilization of Multiple Recycled Materials in Asphalt Concrete: Mechanical Characterization and Cost–Benefit Analysis

**DOI:** 10.3390/ma17194742

**Published:** 2024-09-27

**Authors:** Heui-Soo Han, Behnam Golestani, Kyungwon Park, Byounghooi Cho, Boo Hyun Nam

**Affiliations:** 1 Department of Civil Engineering, Kumoh National Institute of Technology, Gumi 39177, Republic of Korea; hanhs@kumoh.ac.kr; 2 Professional Service Industries, Inc. (PSI), 1748 33rd St., Orlando, FL 32839, USA; ben.gol@intertek.com; 3 Department of Civil Engineering, College of Engineering, Kyung Hee University, Yongin 17104, Republic of Korea; kwpark@khu.ac.kr; 4 Department of Civil Engineering, Sangmyung University, Cheonan 31066, Republic of Korea; byoungcho@smu.ac.kr

**Keywords:** municipal solid waste incineration (MSWI) bottom ash (BA), recycled asphalt shingle (RAS), recycled concrete aggregate (RCA), life cycle cost analysis (LCCA)

## Abstract

This study examines the strategic incorporation of various recycled materials into asphalt concrete, specifically focusing on municipal solid waste incineration bottom ash (MSWI BA), recycled asphalt shingle (RAS), and recycled concrete aggregate (RCA). Due to the high porosity of MSWI BA and RCA, and the significant asphalt binder content (30–40%) found in RAS, there is a need to increase the amount of liquid asphalt used. RAS is posited as an efficient substitute for the asphalt binder, helping to counterbalance the high absorption characteristics of MSWI BA and RCA. The research objective is to quantitatively evaluate the effect of the combined use of RAS, MSWI BA, and RCA in Hot Mix Asphalt (HMA). This study encompasses several laboratory evaluations (i.e., rutting and tensile strength tests) and a cost–benefit analysis, which is a life cycle cost analysis. The results indicate that the combined use of these materials results in a higher tensile strength and rut resistance when compared with the control (with virgin aggregate). According to the cost–benefit analysis result, when the three recycled materials are used for an HMA overlay over an existing aged pavement, it could be 60–80% more cost-effective compared to a conventional HMA overlay, thereby offering significant economical savings each year in the field of road construction.

## 1. Introduction

The global population increase has escalated waste generation, intensifying the strain on landfill capacities and amplifying the costs associated with waste disposal. In response, substantial progress has been made toward implementing sustainable material practices, notably through the recycling of waste materials in diverse engineering applications [1]. As environmental concerns mount alongside rising energy costs, the demand for sustainable, recyclable engineering materials has surged. Significant material resources are required for road construction, and this is an area where these sustainable materials can be innovatively utilized. For example, high-quality fractions of soil-rock mixtures can serve as aggregates in asphalt or concrete, medium-quality fractions can fill road embankments, and low-quality parts can be stabilized for use in roadbeds. Waste materials are often described as “resources in the wrong places”, highlighting their potential for recycling or reuse. In regions such as Europe and the U.S.A., solid waste materials (or recycled construction materials) are categorized based on their origins as industrial, road, or demolition byproducts [2]. Replacing virgin aggregate with recycled materials is an evolving practice, aimed at conserving energy and natural resources, reducing landfill use, and mitigating the adverse impacts associated with the extraction and transportation of virgin materials [3].

The role of municipal solid waste incineration bottom ash (MSWI BA) in waste management is critical. As MSWI BA is a byproduct of municipal solid waste incineration at combustion facilities, the strategic management of MSWI ashes is increasingly recognized worldwide. Several countries have adopted strategic management plans and regulations to promote the beneficial use of MSWI ashes [4,5,6,7,8,9]. In Europe, MSWI bottom ash is utilized in sustainable transport projects, compliant with the environmental standards established through regulatory frameworks [8,9,10]. Despite its high waste output, the United States exhibits a relatively low recycling rate [11,12]. Since 1980, MSW production in the United States has increased by 65%, reaching an annual total of 250 million tons. Of this, 53.6% is disposed of in landfills, 34.7% is recycled or composted, and 11.7% is incinerated for energy recovery. Additionally, about 10% of MSWI BA is currently used in the area of road construction. There have been research efforts to utilize MSWI BA in asphalt concrete beneficially, and also to optimize mix proportioning for the best mechanical performance [13,14,15].

Since the mid-1970s, the United States has initiated practical measures to utilize recycled materials, including using reclaimed asphalt pavement (RAP) and recycled asphalt shingle (RAS) in road construction projects. RAS contains a significant amount of aged asphalt binder (30–40%) and has been reused in road materials [16,17]. RAP is particularly valued for its cost-effectiveness and minimal environmental impact, and is capable of partially substituting both asphalt binder and aggregate. The Illinois DOT utilized approximately 1.7 million tons of waste materials in the state’s highway projects in 2010 [18].

Recycled concrete aggregate (RCA) is derived from demolished concrete, is produced by the process of crushing and sieving, and is reused as aggregate in either roadbed or concrete [19,20,21,22,23,24,25]. Initially, RCA was primarily landfilled; however, extensive research [26,27,28,29,30,31,32,33] has enabled its use as a road subbase material and in nonstructural concrete applications. RCA differs from virgin aggregates primarily due to the cement paste adhering to the aggregate surface, making it highly porous and prone to higher water absorption [34,35,36,37,38,39,40,41]. This high porosity necessitates an increased binder absorption, making higher substitution ratios economically challenging. A number of researchers have investigated the impact of RCA in HMA. Sumeda et al. (2006) [1] reported that the use of RCA in HMA reduces bulk density and film thickness in the asphalt mixture. Another study by Beale et al. (2009) [42] showed that the partial replacement of RCA in HMA decreases the dynamic stiffness of the mixture when compared with the control mix; thus, the study recommended the use of RCA for low-volume roads. Most previous studies have focused on investigating the effects of an individual recycling material in pavement, typically evaluating the mechanical behavior of the asphalt mixture using laboratory testing methods. The presented study not only investigates the effects of multiple recycled materials that compensate for each other in the mixture, but also quantitatively conducts a cost–benefit analysis using a life cycle cost analysis (LCCA).

The main objective of this study is to explore the combined use of three recycled materials in HMA, and to identify their optimum combination and proportioning. In this configuration, RAS serves as an additive, MSWI BA replaces fine aggregate, and RCA substitutes for coarse aggregate. Following the insights from Nam et al. (2023) [43], which demonstrated enhanced performance with a 20% substitution of virgin fine aggregates with MSWI BA, this specific mixture ratio was maintained across all samples, while preserving consistent gradation. The substitution percentages utilized were 20% for BA and 100% for RCA, with RAS content varying from 0% to 6%. All samples were prepared in accordance with Superpave Mix Design standards. The mechanical performance of the mixtures was quantified through tensile strength and rutting tests. Additionally, the economic viability of using these recycled materials was analyzed through a cost–benefit evaluation, namely a life cycle cost analysis (LCCA).

## 2. Experimental Study

This paper is intended as an academic discussion, not as engineering advice, and no reliance upon this paper is permitted. Independent advice by the professional of record as to the application of the concepts and opinions herein to any specific project should be sought.

### 2.1. Materials

#### 2.1.1. Binder

The asphalt binder, PG 67-22, was used in this study. This binder is stiff and commonly applied in Florida’s road constructions. It was sourced from a local provider in Bradenton, Florida. The physical properties of the asphalt binder are summarized in Table 1.

#### 2.1.2. Aggregate

This study utilized three different types of aggregates: Limerock, MSWI BA, and RCA. The fundamental physical properties of these aggregates and their testing protocols are listed in Table 2.

### 2.2. Sample Preparation

**Figure 1:**
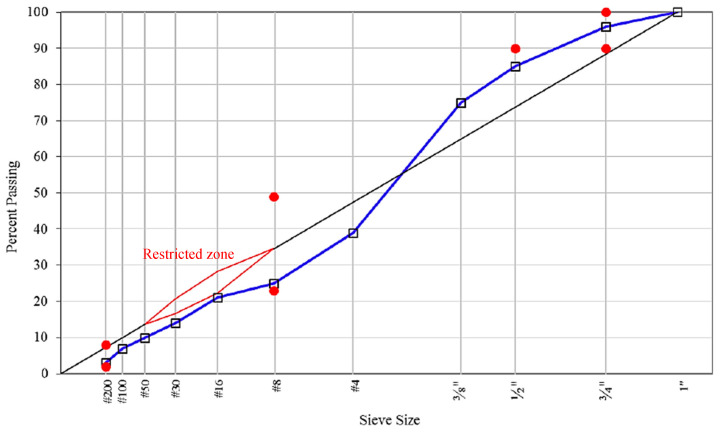
Particle distribution curve of aggregate along with Superpave’s gradation requirement. (note: red = control points, blue line = design aggregate structure, black line = maximum density line).

The determination of the optimal asphalt content (OAC) was achieved using the Superpave Mix Design method. For the control mix, which utilized conventional asphalt, the OAC was established at 5.1%, targeting an air void content of 4%. The aggregate used in HMA meets the gradation requirements of the Superpave Mix Design, as shown in Figure 1. Each sample was assigned a unique code to facilitate identification and analysis, as shown in Table 3. To explore the effects of RCA, it was used to replace the coarse aggregates in the control mix, which was originally composed of virgin aggregates, at the replacement rates of 25%, 50%, 75%, and 100% by weight. The control mix OAC was maintained across all mixtures.

Our previous study, Nam et al. (2023) [42], confirmed that a 20% replacement of fine aggregate with MSWI BA exhibits the most improved mechanical performance; thus, the scope is to limit the MSWI BA to a 20% replacement of fine aggregate in HMA. This section highlights the approach of utilizing HMA with 100% replacement of coarse aggregate with RCA and a 20% substitution of fine aggregate with MSWI BA. RAS was added to compensate for the increased demand for asphalt binder due to the high porosity of RCA and MSWI BA. The use of RAS as an additive ranged from 1% to 6% by the total mass of the aggregate.

### 2.3. Testing Procedure

#### 2.3.1. Indirect Tensile Strength Test

Existing research has extensively documented the influence of tensile strength on pavement performance through the study of crack propagation in pavements [46,47,48]. When determining the fatigue life of HMA, the tensile stress/strain at the bottom of the HMA layer is an important criterion. In HMA, fatigue cracks typically initiate at the bottom, and then propagate upwards under repeated vehicle loading. This can lead to fatigue cracks and, eventually, to the formation of bottom-up reflective cracks.

**Figure 2:**
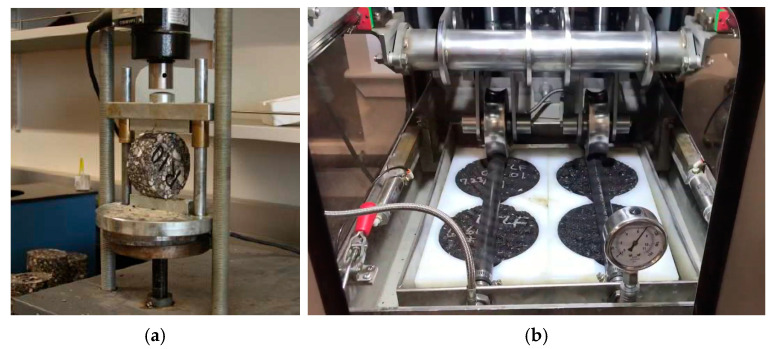
Laboratory testing methods: (**a**) indirect tensile test (IDT), (**b**) asphalt pavement analyzer (APA) test.

This study measured indirect tensile strength using the indirect tensile test (IDT) apparatus (see Figure 2a), applying a loading rate of 2 inches per minute. The test followed the procedures described in ASTM D4867 [49]. Each set of specimens was tested at a temperature of 25 °C. The dry samples were hermetically sealed and submerged in a water bath to maintain a thermal equilibrium of 25 °C. The calculation formula is as follows:

S_t_ = 2000 P/πtD (kPa) (1)

where S_t_ = tensile strength [kPa], P = maximum load [N], t = specimen height immediately before the tensile test [mm], and D = specimen diameter [mm].

#### 2.3.2. Rutting Test

The rutting characteristics of each mixture were evaluated using a rut testing method, specifically an asphalt pavement analyzer (APA). Figure 2b shows the APA apparatus and the testing, followed by AASHTO T340. This test is crucial for determining the stiffness of the mix. The results are straightforward because the rut depth of each specimen was directly measured as the moving load was repeated. The experiments were conducted on 75 mm dry HMA specimens (cylinders with 7.0 ± 0.5 percent air voids), prepared by a Superpave Gyratory Compactor (SGC) with 8000 loading cycles at 64 degrees Celsius. The rut depth is measured upon the completion of the 8000 load cycles.

## 3. Results

### 3.1. Tensile Strength

**Figure 3:**
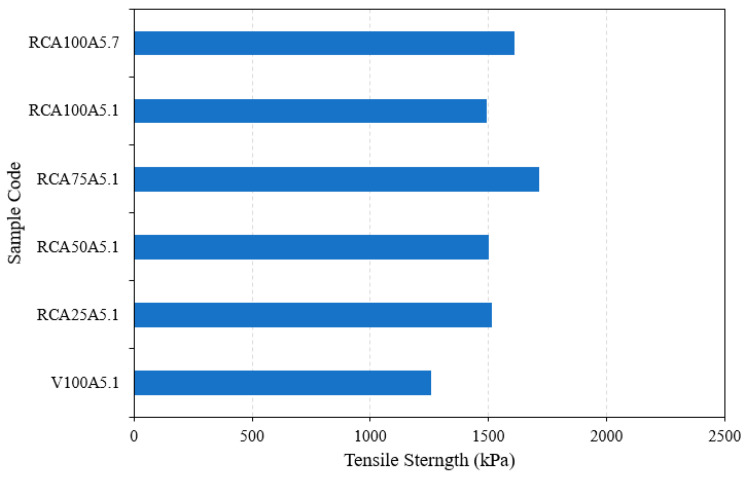
Results of the IDT for the specimens containing RCA only.

**Figure 4:**
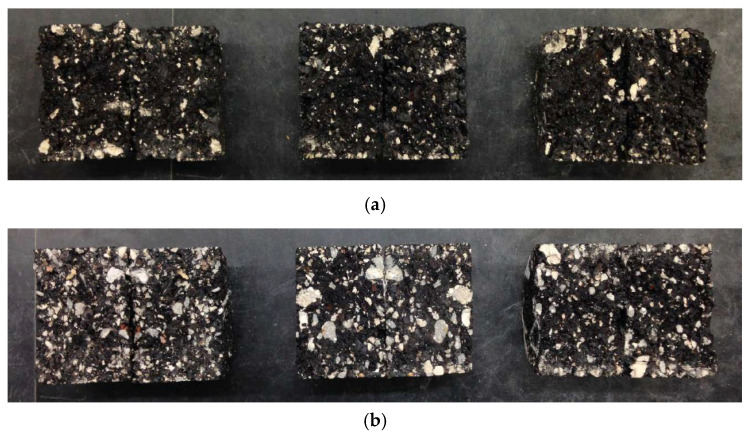
Fractured surface after IDT: (**a**) virgin aggregate (sample ID: ‘HMA V100A5.1’), (**b**) RCA (sample ID: ‘HMA RCA100A5.1’).

Figure 3 illustrates the effect of substituting coarse aggregate with Recycled Concrete Aggregate (RCA) in Hot Mix Asphalt (HMA). The graph shows tensile strength data for HMA mixtures with varying levels of RCA substitution at 25%, 50%, 75%, and 100%. The increased number of fractured surfaces and enhanced surface texture in RCAs contribute to better adhesion, which is reflected in the rising trend in tensile strength, peaking at 75% replacement. Figure 4a,b display examples of fractured HMA samples after testing the tensile strength, highlighting the superior aggregate–binder adhesion in mixes with RCA when compared to those with virgin aggregates, as seen in Figure 4. A decrease in tensile strength at 100% was observed, likely due to a lack of sufficient asphalt binder and poor asphalt coating within the mix, a finding that supports the need to increase the asphalt content to an optimal asphalt content (OAC) of 5.7%.

**Figure 5:**
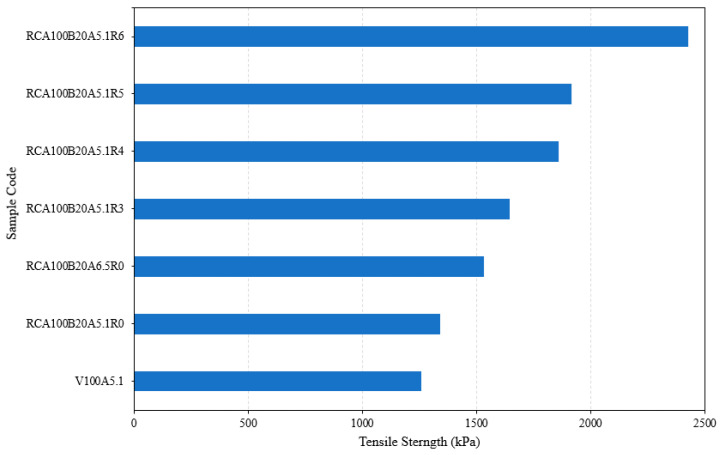
Results of the IDT for the specimens containing RCA (100% replacement), MSWI BA (20% replacement), and varied RAS content.

An additional sample was prepared by increasing the asphalt binder content to 5.7%, while maintaining a 100% replacement of RCA. The results from the indirect tensile test (IDT) revealed a significant improvement in tensile strength, with an increase of 115 kPa. To optimize the use of RCA, a 20% replacement of the original fine aggregates with bottom ash (BA) was implemented in the mix designated “RCA100A5.1”. Consequently, the OAC increased to 6.5%. This 1.4% increase in OAC may not align with the study’s goal of conserving resources. Thus, alternative proportions of recycled asphalt shingle (RAS) were integrated into the mix “RCA100B20A5.1”, and the outcomes are depicted in Figure 5. The tensile strength improved as expected when the asphalt content reached the OAC in the sample “RCA100B20A5.1R0”. The addition of RAS, and the subsequent increase in asphalt content, resulted in increased binder viscosity, thereby enhancing tensile strength.

### 3.2. Rutting Resistance

**Figure 6:**
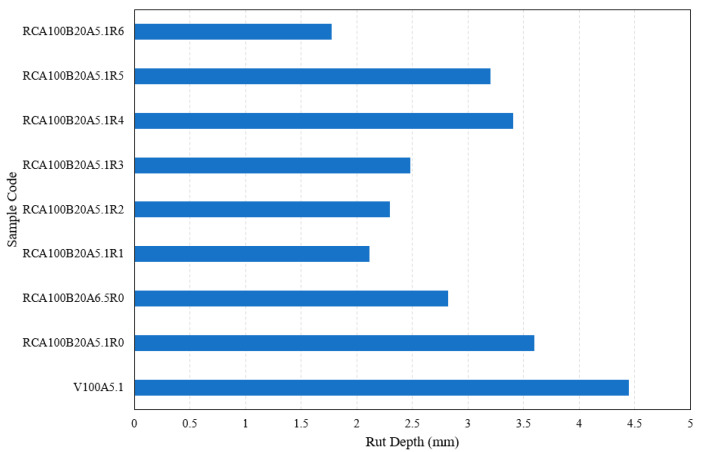
Results of the APA test for the specimens with RCA (100%), BA (20%), and varied RAS content.

The rutting resistance was evaluated using APA testing. In APA testing, 8000 loading cycles are applied and the rut depth is measured upon their completion, followed by AASHTO T340. The results, shown in Figure 6, indicate that unlike the tensile strength, the rutting resistance does not show consistency, such as either an increasing or decreasing tendency with increasing RAS content. However, it is obvious that RAS 1–3% shows a lower rut depth than RAS 4–6%.

This observation may stem from variations in effective binder content (EBC) and the aggregate’s properties. Compared with the control mixture with no RCA (“V100A5.1”), the mixtures with RCA show much lower rut depths. The angularity and rough texture of RCA may contribute to strong interlock in the mixture “RCA100B20A5.1R0”, enhancing resistance to permanent deformation when binder content is increased to the optimum asphalt content (OAC). A notable increase in EBC was observed in the mixes with 5% and 6% RAS, where the effect of EBC on rutting resistance is more pronounced.

### 3.3. IDT Stiffness

**Figure 7:**
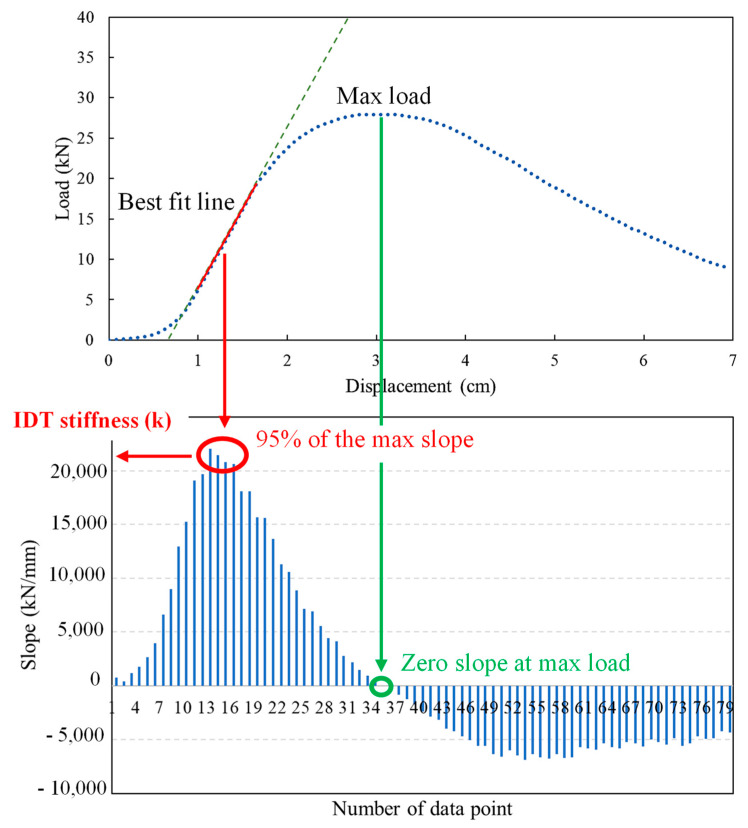
Determination of the material stiffness (k value) from IDT.

Although the IDT provides a measure for the indirect tensile strength, the slope of the load–displacement curve also indicates the stiffness of asphalt mixtures. The slope index, derived from the slope of the load–displacement curve of the IDT, is represented as “IDT stiffness (*k*)”. Steeper slopes suggest stiffer materials, and generally correlate with better rutting resistance. Figure 7 illustrates the computation procedure to determine the IDT stiffness (*k*). An algorithm was developed and implemented in MATLAB (Nam et al., 2023) [42].

**Figure 8:**
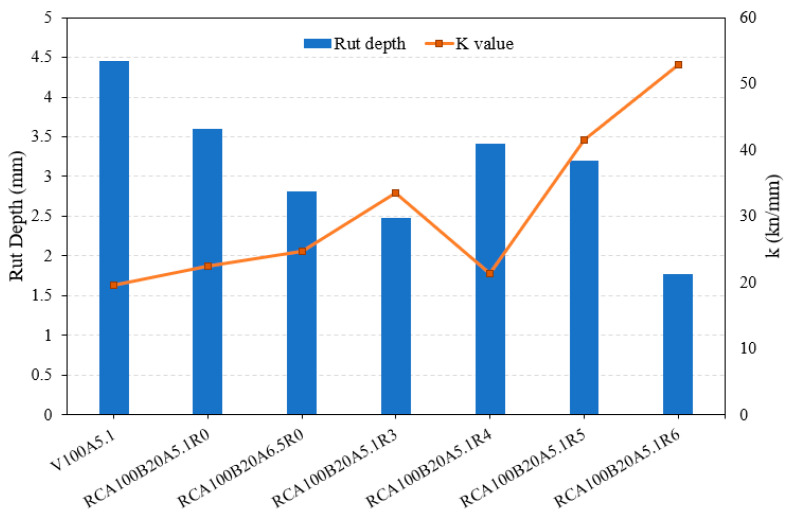
Comparison between rut depth and IDT stiffness (*k* value) for RCA-BA-RAS mixtures.

Figure 8 presents the relationship between the “*k*” value and rut depths, demonstrating a clear pattern where higher “*k*” values coincide with reduced rut depths. The correlation between rutting resistance and material stiffness (load–displacement) was established for all mixtures and then compared to each other. As seen in the figure, the overall relationship between IDT stiffness and rut depth seems to be inversely proportional. The control (V100A5.1) has the largest rut depth, but the lowest *k* value. As RAS increases, the rut depth of the mixtures decreases, probably due to the aged binder in RAS, and the *k* value increases with increasing RAS content.

A statistical correlation between the IDT stiffness and rut depth was identified using the “Pearson product-moment correlation coefficient (PCC)”. It is noted that the PCC measures the linear correlation between two variables, ranging from −1 to +1. The calculated mean value is −0.78, which indicates a strong inverse correlation between the two results.

## 4. Cost–Benefit Analysis

### 4.1. Life Cycle Cost Analysis (LCCA)

This section involves performing an LCCA to evaluate the cost-effectiveness of the proposed asphalt mixtures. LCCA is an essential tool for assessing the long-term financial viability of projects, capturing both the initial and future anticipated costs throughout a project’s lifetime. It serves as a crucial decision-making tool by providing a comprehensive economic analysis of various investment scenarios to stakeholders. This analysis includes all relevant costs, for example initial construction, maintenance and rehabilitation, resurfacing, and reconstruction, which are incurred during a project’s life span.

It is known that the Federal Highway Association (FHWA) promotes LCCA as a tool for choosing the most economically advantageous option, and for communicating with the public to ensure the best choice is made [50]. LCCA should incorporate discounted agency costs, user costs, and other costs over the performance period of the pavement project [51,52,53,54]. The selection of an appropriate discount rate, typically between 3% and 5%, significantly influences LCCA results. User costs include expenses incurred by drivers due to construction-related detours, and while these should be calculated differently for each scenario to avoid nullifying comparisons, they must still be documented.

In this study, an a cost-effectiveness analysis of an asphalt concrete (AC) overlay over 20 years of service life was conducted. Due to the absence of field data for AC overlay’s performance with the proposing recycled materials (i.e., RCA, BA, RAS), laboratory testing results were used to evaluate and predict the maintenance frequency and the service life of the overlay when these recycled materials are used. Critical performance indicators, such as IDT strength, rut depth, and fracture energy, which help gauge the potential for cracking and deformation in HMA, were key to this estimation. Table 4 presents the expected service life of asphalt overlays when various waste materials are integrated, using both linear and variable impact analyses.

The information provided in Table 5 presents the pavement description for the four possible combinations, considering a two-inch-thick AC overlay and a 5-year service life.

Table 6 displays the components of the LCC. The costs are denominated in US dollars. The prices displayed are the mean values obtained from various states. In order to account for the uncertainty in the data about the cost of asphalt binder and the tipping charge for landfilling, Monte Carlo simulation was utilized.

### 4.2. LCCA Result

The financial analysis was conducted by estimating the cost savings achieved through the use of recycled concrete aggregate (RCA), bottom ash (BA), and recycled asphalt shingle (RAS) in various scenarios. The differences in material service life affect the amount of material required over the pavement’s lifespan. Table 7 details the cost calculations for the proposed mixes, considering a single-lane road stretching one mile with a two-inch overlay applied at varying maintenance intervals over 20 years. The total volume of the pavement overlay was consistently estimated at 302.49 cubic meters across all scenarios. The density and mass of the materials, crucial for determining the quantities of recycled and virgin materials needed, are documented in Table 4 and Table 5.

**Figure 9:**
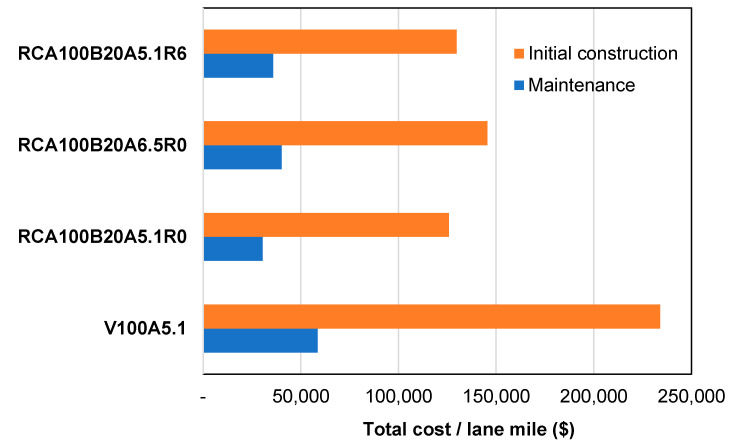
Total cost per lane mile for a 20-year service life.

Figure 9 shows the LCCA result, summarizing the total cost per lane mile for a 20-yr service life. It is important to note that RCA, RAS, and BA are treated as byproducts, thereby incurring no manufacturing costs. However, this study incorporates the landfill tipping fees associated with disposing of these recycled materials, a factor that significantly affects the overall financial analysis. Ultimately, the total cost of constructing and maintaining an overlay using the HMA mix “RCA100B20A5.1R6” is found to be 60–80% lower than that of an overlay using new materials. This considerable cost reduction highlights the potential for substantial annual savings in pavement construction projects.

## 5. Discussion

This study investigated a limited aspect of the mechanical properties of mixtures of asphalt concrete, but investigated only the most basic properties, those of tensile strength and rut resistance. The measured properties of the specimens were used to estimate their field performance compared with a control specimen. However, for a more accurate prediction of the field performance, a more comprehensive laboratory testing program is necessary, and should include durability performance under severe environmental conditions (e.g., freeze/thaw), fatigue performance (against fatigue crack), and so on. As seen in Figure 8, the mixtures exhibit an inversely proportional relationship between the IDT stiffness and rut depth. As the rut depth decreases, which is positive, the material becomes more brittle, and is then more susceptible to fracture/cracking. Thus, a more sophisticated study on crack resistance and fatigue damage is recommended for future research. In addition, the combined use of RAS, MSWI BA, and RCA may be implemented in the field, and as such, the long-term monitoring of their field performance is recommended. In the presented study, the scope of the work was limited to the feasibility and cost-effectiveness of the combination of the three recycled materials in an asphalt mixture.

## 6. Conclusions

This study investigated the mechanical properties of an asphalt mixture and conducted a financial assessment on the asphalt mixture, incorporating varying ratios of three recycled materials: RCA, MSWI BA, and RAS. In this study, RCA and MSWI BA replaced virgin aggregate, while RAS was used as an additive. Laboratory testing was used to evaluate the mechanical performance of the mixtures, and an LCCA was used to determine the level of cost–benefit effectiveness over their service life. The key outcomes from both the laboratory experiments and the economic analysis are summarized below:

- The distinct porosity and uneven surface texture of MSWI BA enhance the asphalt binder absorption. Replacing 20% of fine aggregates with MSWI BA increases the optimum bitumen content by 1.1%, improving the stiffness and tensile strength, due to better aggregate interlocking.
- During mixing or compaction, MSWI BA particles might break down, creating more filler material and potentially reducing air voids. Therefore, adjustments in gradation should be considered in the mix design process to address these changes.
- The rutting test results indicate a negative correlation between the effective asphalt binder content and the rutting resistance, with mixes below the optimal asphalt content (OAC) showing deeper ruts. While RAS increases the mix stiffness, excessive RAS can lead to increased plastic deformation and greater rut depth.
- The integration of 6% RAS, 100% RCA replacement for coarse aggregate, and 20% MSWI BA substitution for fine aggregate in HMA can reduce construction and maintenance costs by 60–80% over 20 years, representing substantial economic benefits.

## Figures and Tables

**Table 1:** Physical properties of the asphalt binder.

Test	Test Method	Specification	Test Results
Rotational viscosity @ 135 °C, 20 rpm spindle # 21	T316	3.0 Max	0.465 Pa.s
Rotational viscosity @ 165 °C, 20 rpm spindle # 21	T316	3.0 Max	0.128 Pa.s
Dynamic shear (G*/sin δ, 10 rad/s)	T315	1.0 min @ 67 °C	1.09 kPa
Ring and ball soft point	T53	-	54 °C
Penetration @ 25 °C	T49	-	59 dmm
Flash point	T48	230 °C	344 °C

Note: Pa.s = pascal = −second.

**Table 2:** Physical properties of the coarse aggregates.

Properties	Limestone	BA	RCA	Test Methods
Specific gravity (oven dry)	2.4 *	-	2.19	ASTM C127 [43]
Absorption capacity, %	3.04	12.8	6.45	ASTM C127 [43]
% of fractured particles in coarse aggregates (1 fractured face/2 fractured face)	81.4/74.7	N/A	88.7/83.24	ASTM D5821 [44]
L.A. abrasion mass loss, %	36.5	43	41.3	ASTM C131 [45]

* Fine aggregate specific gravity is 2.5.

**Table 3:** Sample codes and descriptions of mixture specimens.

Sample Codes	Description
V100A5.1	100% virgin coarse and fine aggregates @ 5.1% OAC
RCA25A5.1	25% RCA coarse + 100% virgin fine @ 5.1% AC
RCA50A5.1	50% RCA coarse + 100% virgin fine @ 5.1% AC
RCA75A5.1	75% RCA coarse + 100% virgin fine @ 5.1% AC
RCA100A5.1	100% RCA coarse + 100% virgin fine @ 5.1% AC
RCA100A5.7	100% RCA coarse + 100% virgin fine @ 5.7% OAC
RCA100B20A5.1	100% virgin coarse + 80% virgin fine + 20% BA fine + 0% RAS@ 5.1% AC
RCA100B20A5.1R1	100% virgin coarse + 80% virgin fine + 20% BA fine + 1% RAS@ 5.1% AC
RCA100B20A5.1R2	100% virgin coarse + 80% virgin fine + 20% BA fine + 2% RAS@ 5.1% AC
RCA100B20A5.1R3	100% virgin coarse + 80% virgin fine + 20% BA fine + 3% RAS@ 5.1% AC
RCA100B20A5.1R4	100% virgin coarse + 80% virgin fine + 20% BA fine + 4% RAS@ 5.1% AC
RCA100B20A5.1R5	100% virgin coarse + 80% virgin fine + 20% BA fine + 5% RAS@ 5.1% AC
RCA100B20A5.1R6	100% virgin coarse + 80% virgin fine + 20% BA fine + 6% RAS@ 5.1% AC

**Table 4:** Estimated service life.

Sample Codes	IDT Strength (kPa)	Rut Depth (mm)	Fracture Energy (N.mm)	Service Life (yrs.)
Impact Assessment	30%	40%	30%	100%
V100A5.1	1263.25	4.450	43855.22	5
RCA100B20A5.1R0	1345.3	3.599	39437.89	5.54
RCA100B20A6.5R0	1533.70	2.819	48323.2	6.33
RCA100B20A5.1R6	2435.50	1.774	43296.9	6.28

**Table 5:** Pavement description of the four possible combinations.

Sample Code	Length (mi)	Width (ft)	Surface Depth (in)	Base Depth (in)	RAP Removal (in)	Density (ton/m^3^)	Overlay Mass (tons)	Maintenance Frequency
V100A5.1	1	12	2	0	2	2.09	632.21	4
RCA100B20A5.1R0	1	12	2	0	2	2.03	614.06	3.61
RCA100B20A6.5R0	1	12	2	0	2	2.045	618.60	3.16
RCA100B20A5.1R6	1	12	2	0	2	2.06	623.13	3.18

**Table 6:** LCC components.

LCC Components	Unit	Value	References
Virgin Aggregate	USD/ton	USD 50	[50]
Sand	USD/ton	USD 40	[50]
Asphalt Binder	USD/ton	USD 505–USD 697	[55,56,57]
Trucking	USD/ton/mile	USD 0.13	[58]
Tipping Fee	USD/ton	USD 24.3–USD 91	[59]
Shingle Grinding	USD/ton	USD 14.80	[50]
Asphalt Inflation Rate	%/year	%1.1	[60,61]
Trucking Distance [Mine to Plant]	Miles	30	[50]
Trucking Distance [Refinery to Plant]	Miles	50	[50]
Trucking Distance [Plant to Site]	Miles	10	[50]

**Table 7:** Calculation of the cost (initial construction and maintenance) for the four proposed mixtures.

HMA Code		V100A5.1	RCA100B20A5.1R0	RCA100B20A6.5R0	RCA100B20A5.1R6
**Initial construction**	VA Cost	USD 25,797.00	USD 5007.65	USD 4701.34	USD 5081.66
	Sand Cost	USD 3360.00	USD 3264.34	USD 3241.45	USD 3312.58
	Binder Cost	USD 19,057.00	USD 18,510.30	USD 23,765.89	USD 18,783.86
	VA Trucking	USD 1950.00	USD 378.58	USD 355.42	USD 384.17
	RCA Trucking	-	USD 1343.94	USD 1353.87	USD 1363.80
	Sand Trucking	USD 317.00	USD 308.48	USD 306.32	USD 313.04
	Binder Trucking	USD 203.00	USD 197.30	USD 253.32	USD 200.21
	BA + RAS Trucking	-	USD 171.76	USD 170.70	USD 315.63
	Plant-to-Site Trucking	USD 7960.00	USD 773.71	USD 779.43	USD 785.15
	RCA Tipping Credit	-	USD 522.89	USD 5261.48	USD 5300.07
	**Total**	**USD 58,644.00**	**USD 30,478.95**	**USD 40,189.22**	**USD 35,840.17**
**Maintenance**	Sand Cost	USD 117,242.00	USD 2055.99	USD 16,971.96	USD 18,482.90
	Binder Cost	USD 15,274.00	USD 13,399.83	USD 11,701.72	USD 12,048.45
	VA Trucking	USD 86,611.00	USD 75,983.21	USD 85,795.58	USD 68,320.25
	RCA Trucking	USD 8863.00	USD 1554.03	USD 1283.08	USD 1397.31
	Sand Trucking	-	USD 5516.77	USD 4887.52	USD 4960.40
	Binder Trucking	USD 1443.00	USD 1266.28	USD 1105.81	USD 1138.58
	BA + RAS Trucking	USD 923.00	USD 809.89	USD 914.48	USD 728.21
	RCA Tipping Credit	-	USD 705.08	USD 1038.28	USD 633.97
	BA Tipping Credit	USD 3620.00	USD 3176.03	USD 2813.77	USD 2855.73
	RAS Cost	-	USD 21,439.51	USD 18,994.11	USD 19,277.32
	**Total**	**USD 233,976.00**	**USD 125,906.62**	**USD 145,506.31**	**USD 129,843.12**

## Data Availability

Data are contained within the article.
